# Reduced chemical shift-induced phase errors at 3T using novel PC-MRI encoding gradients

**DOI:** 10.1186/1532-429X-15-S1-W35

**Published:** 2013-01-30

**Authors:** MJ Middione, D Ennis

**Affiliations:** 1 Department of Radiological Sciences, University of California, Los Angeles, CA, USA; 2 Biomedical Physics Interdepartmental Program, University of California, Los Angeles, CA, USA

## Background

Cardiovascular MRI benefits from improved SNR-efficiency at ≥3T [1,2], but is subject to other sources of error, which require careful consideration when transitioning from primary use of 1.5T scanners. For example, chemical shift-induced PC-MRI errors [3] are increased at 3T compared to 1.5T. Chemical shift causes the complex perivascular fat signal to chemically shift into the vessel lumen and superposes with the complex blood (water) signal, thereby corrupting the phase (velocity) estimate. Chemical shift errors can be minimized by increasing the bandwidth (reduces the magnitude of the shifted fat signal), and by using an in-phase TE (TE_IN_, ensures fat and water are in-phase). Shorter TEs improve SNR, therefore it is advantageous that the minimum TE_IN_ (TE_IN,MIN_) at 3T is 2.46ms, which is substantially shorter than TE_IN,MIN_=4.92ms at 1.5T, but such short TEs cannot be attained with conventional flow-compensated/flow-encoded (FCFE) velocity encoding strategies. The ***objective*** was to design a velocity encoding strategy void of conventional FCFE gradients that instead achieves through-plane velocity sensitivity using the slice select gradient, which yields a non-zero first gradient moment (M_1_) for the first PC-MRI TR: M_1,1_=X. The slice-select refocusing gradient (SSRG) lobe is time-shifted for the second TR to produce M_1,2_=X+Y, such that ΔM_1_=Y=π•γ^-1^•VENC^-1^. We ***hypothesize*** that the proposed SSRG velocity encoding scheme, will permit the use of TE_IN,MIN_ for chemical shift insensitive PC-MRI measures at 3T that are both faster and have improved SNR.

## Methods

PC-MRI measurements were acquired in volunteers (N=10) on a Siemens 3T scanner with SSRG: TE/TR=2.46/4.46ms (TE_IN,MIN_), 192×132 matrix, 1.6mm^2^×6mm resolution, 30° flip angle, 814Hz/pixel (high bandwidth, HBW), 4 views-per-segment, 35.7ms temporal resolution, and VENC=200cm/s. 2D through-plane velocity encoding was acquired in the ascending aorta (aAo), main pulmonary artery (PA), and right and left pulmonary arteries (RPA and LPA). For comparison FCFE PC-MRI was acquired with the following changes: TE/TR=3.08/6.04ms (TE_MID_), 401Hz/px (low bandwidth, LBW), and 48.3ms temporal resolution. Eddy current background phase errors were corrected [4]. Intra-subject flow agreement (flow difference between vessels) was compared for SSRG and FCFE for all vessel pairs (aAo vs. PA, aAo vs. RPA+LPA, and PA vs. RPA+LPA).

## Results

**Figure 1:**
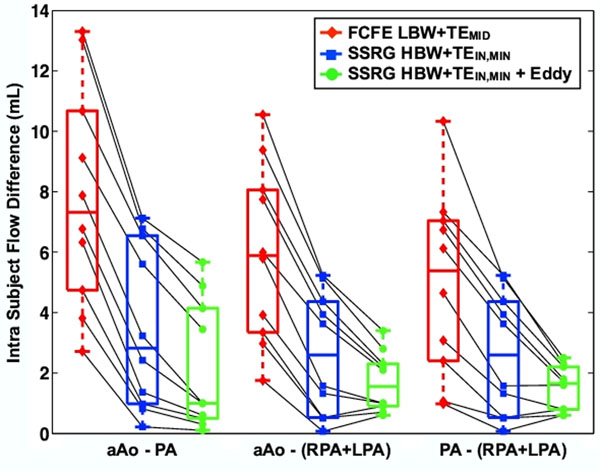
Intra-subject flow agreement between the conventional flow compensated and flow encoded (FCFE) velocity encoding sequence at LBW+TE_MID _**(red diamonds)** and the slice select refocusing gradient (SSRG) sequence at HBW+TE_IN,MIN_ without **(blue squares)** and with **(green circles)** eddy current correction. Data from individual subjects (N=10) are connected to show that SSRG with HBW+TE_IN,MIN_ leads to better intra-vessel flow agreement for every subject compared to FCFE with LBW+TE_MID_. Correcting for eddy currents further improves the agreement. The box plot shows the median and 25th and 75th percentiles and the error bars show the 95% confidence intervals.

Figure 1 and Table 1 show significantly improved intra-subject flow agreement for SSRG compared to FCFE. SSRG also provides a 26% increase in temporal resolution and a 33% increase in SNR (87.7±40.8 vs. 58.5±24.8, P=0.01) compared to FCFE.

**Table 1:** Intra-subject percent flow difference from the pre-clinical evaluation of ten normal volunteers (N=10) expressed as a mean ± SD [minimum, maximum].

	FCFE LBW+TE_MID_	SSRG HBW+TE_IN,MIN_	*P-Value
aAo vs. PA	5.8 ± 2.8% [0.98, 8.9%]	1.7 ± 1.9% [0.16, 2.8%]	0.002

aAo vs. RPA+LPA	6.0 ± 4.3% [0.85, 9.8%]	2.1 ± 1.7% [0.60, 2.5%]	0.03

PA vs. RPA+LPA	6.1 ± 6.3% [0.11, 7.6%]	2.9 ± 2.1% [0.57, 2.2%]	0.04

*P < 0.05 show a statistical significant difference between FCFE LBW+TE_MID_ and SSRG HBW+TE_IN,MIN_ indicating significant improvement in internal consistency flow measures using SSRG HBW+TE_IN,MIN_ in PC-MRI.

## Conclusions

Our 3T optimized SSRG PC-MRI sequence minimizes chemical shift-induced phase errors and improves intra-subject flow agreement compared to FCFE.

## Funding

This work is supported in part by NIH/NHLBI K99-R00 HL-087614 and Siemens Medical Solutions.

## References

[B1] Lotz JMRI 2005

[B2] Strecker JMRI 2012

[B3] Middione MRM 2012

[B4] Chernobelsky JCMR 2007

